# Psychometric Properties of the Perfectionism Cognitions Inventory in Ecuador

**DOI:** 10.3390/ijerph17165834

**Published:** 2020-08-12

**Authors:** María Pilar Aparicio-Flores, María Vicent, Ricardo Sanmartín, Carolina Gonzálvez, Roberto Ovidio Freire-Andino, José Manuel García-Fernández

**Affiliations:** 1 Department of Developmental Psychology and Teaching, Faculty of Education, University of Alicante, 03690 Alicante, Spain; pilar.aparicio@ua.es (M.P.A.-F.); ricardo.sanmartin@ua.es (R.S.); carolina.gonzalvez@ua.es (C.G.); josemagf@ua.es (J.M.G.-F.); 2 Faculty of Social Communication, Central University of Ecuador, Quito 170521, Ecuador; rofreire@uce.edu.ec

**Keywords:** perfectionistic automatic thoughts, validation, factorial invariance, latent mean differences, PCI

## Abstract

Perfectionistic Automatic Thoughts (PATs) are currently being studied due to their association with maladaptive variables. This study aims to validate the Spanish version of the Perfectionism Cognitions Inventory (PCI) in a sample of Ecuadorian undergraduates as well as to analyze latent mean differences across sex. The sample was composed by 3060 undergraduates (*M*_age_ = 22.7, *SD* = 2.46). The Spanish model of the PCI composed by 17 items divided into three first-order dimensions (perfectionistic concerns, strivings, and demands) and a second-order factor was supported by confirmatory factor analysis. Acceptable levels of reliability and factorial invariance across sex were observed. Higher latent mean scores for males in comparison with females in the second-order factor of the PCI were found. The three dimensions of the PCI significantly and positively correlated with interpersonal difficulties. Overall, results demonstrate that the Spanish version of the PCI is a valid and reliable measure to evaluate PATs in Ecuadorian undergraduates.

## 1. Introduction

Perfectionism can be defined as a personality characteristic determined by a hypersensitivity to other’s evaluations, self-criticism, concerns about making mistakes, and lack of perfection as well as dichotomous thinking [1]. Perfectionism seems to be strongly linked to rumination because of discrepancies between an individual’s actual self and their ideal self or their actual level of goal attainment and their high ideals [2]. In fact, some authors consider the existence of specific ruminative thoughts of perfectionist nature, i.e., Perfectionistic Automatic Thoughts (PATs), which reflect the need to be perfect as well as worries about being imperfect [3,4]. In consequence, these thoughts emerge when there is a difficulty reaching a goal or when the individual faces stressful situations that demand a perfectionist behavior [5,6]. Their emergence is so spontaneous and repeated that they are associated with ruminant thoughts to such an extent that some authors nickname them perfectionist rumination [7]. Additionally, although PATs have been conceptualized as a state because, assessed considering the context in which it occurs, research has evidenced a certain temporal stability of perfectionist rumination [3,8,9]. In fact, some people apply them in any situation [4] and PATs are well-established cognitions in most perfectionists, being manifested as a characteristic of their own personality [10]. Also, it seems that, even after a year, people with PAT who ruminate on a negative past event take the negative feeling to such an extent that they can experience depressive symptoms a year after having suffered the event [11]. In this sense, research has shown the positive association between PATs and a wide range of psychopathologies, such as depression [12]; emotional distress [3,13,14]; anger, anxiety, and discouragement [15,16]; social anxiety [17,18]; rumination [2,19]; self-criticism, negative thinking, and dependence [19]; psychosomatic symptoms [3,20]; sensitivity to emotional pain [21]; eating behavior disorders [22,23]; and procrastination and fear of failure [24], among others.

Traditionally, research has mainly focused on the study of perfectionism as a personality trait, rather than specifically on PATs. However, these perfectionist ruminations are of great interest for scientific research because they reveal perfectionist cognitions depending on the personal situation of each individual [10], provoking great psychological distress in some cases [2,3,13]. If the model of social disconnection from which perfectionism originates [11,25,26,27] and the generation of negative emotions in the subject are considered in this situation, interpersonal difficulties and their mental state that hinder the link with society can be triggered. Hence, its evaluation is important.

There are few instruments that evaluate PATs. For instance, the *Dysfunctional Attitude Scale* (DAS) [28] assesses two dimensions related to perfectionism and need for approval. However, the DAS items have a clear connotation linked to depressive vulnerability. On the other hand, the *Multidimensional Perfectionism Cognitive Inventory* (MPCI) [29] evaluates perfectionist cognitions across three dimensions: personal standards, pursuit of perfection, and concern over mistakes. However, it has been criticized because the meaning of its items bears a special resemblance to other perfectionist traits that are measured with other more specific scales, such as self-oriented perfectionism. In this regard, the *Perfectionism Cognitions Inventory* (PCI) [3] overcomes all these limitations because it was specifically designed to examine the construct of PATs. 

### Psychometric Properties of the PCI

The PCI was designed by Flett et al. [3] to assess PATs, and it consists of a 25-item self-report scale with a 5-point Likert scale response (1 = *never*; 5 = *always*). Originally, the scale was developed in a sample of 747 Canadian undergraduates (539 females, *M*_age_ = 20.2), obtaining a one-dimensional structure and acceptable reliability (α = 0.96) and temporal stability (*ts* = 0.67) for three-month interval levels. A second [14] and a third analysis [20] of the factorial structure of the PCI performed by the same authors in a clinical sample composed by 258 patients with different psychopathologies (*N* = 134 females) and a community sample composed by 250 Canadian adolescents (*N* = 142 females, *M*_age_ = 15.98) confirmed the original structure of Flett et al. [3]. Moreover, acceptable reliability coefficients were obtained (α = 0.95 and 0.91, respectively, for the clinical and the adolescent sample).

Apart from the three studies performed in Canadian samples by Flett et al. [3,13,19], three other studies have tested, from our knowledge, the psychometric properties of the PCI [18,30,31]. Appleton et al. [30] employed a sample of 190 British junior athletes (73 females, *M*_age_ = 15.2) and added an introduction to all items to adapt them to the sport practice (i.e., *during practice/competition I think*…). With these modifications, the authors found a better adjustment for a one-dimensional 24-item structure, eliminating item number 24 because of its low factor load (0.18). Again, acceptable levels or reliability (α = 0.91) were obtained.

On the other hand, analysis performed by Stoeber et al. [31], using 326 British undergraduates (269 females, *M*_age_ = 19.7), did not support the original one-dimensional 25-item structure of the PCI. Thus, although none of the 25 items were removed, the results evidenced a better adjustment for a three-dimensional structure: Perfectionistic Concerns (PC; defined as recurrent doubts and worries about mistakes, self-ideal discrepancies, and other people’s evaluations; e.g., “Why can’t I be perfect?”); Perfectionistic Strivings (PS; defined as reflections about the excessively high standards; e.g., “My goals are very high.”); and Perfectionistic Demands (PD; defined as automatic thoughts about perfectionistic demands for self-improvement; e.g., “I need to do better.”).

More recently, Esteve-Faubel et al. [18] translated the scale into Spanish and tested by using confirmatory factor analysis both one-dimensional [3] and three-dimensional [31] structures of the PCI proposed by previous research. A sample of 798 Spanish undergraduates was employed (591 females, *M*_age_ = 23.2). Their own model composed by 17 items (by deleting items 6, 8, 10, 11, 19, 20, 21, and 22) structured in three dimensions (PC, PS, and PD, which are, in turn, encompassed in a higher-order dimension) obtained the best adjustment. By this way, the PCI structure purposed by Esteve-Faubel et al. [18] combines the two structures (i.e., one-dimensional and three-dimensional) proposed by previous psychometric studies of the scale.

Due to the lack of consensus about the internal structure of the PCI, the need to expand research about PATs in other non-Western cultures and societies, such as Latin American [18], as well as to test the validity and reliability of the online scale administration, this study aims to analyze the psychometric properties of the Spanish version of the PCI [18] in a sample of Ecuadorian undergraduates. Specifically, it is intended (a) to test the factorial structure of the scale, (b) to analyze its reliability, (c) to examine its factorial invariance across sex as well as (d) latent mean differences as a function of sex, and (e) to study the correlations between the dimensions of the PCI and the different factors that composed the difficulties of social interaction.

## 2. Materials and Methods 

### 2.1. Participants

Participants were selected by proportional random sampling, with faculties as the first level, degrees as the second, semesters as the third, and groups as the fourth, according to different faculties of the Central University of Ecuador in Quito (Ecuador); 3158 participants were contacted, and the objectives of the study were explained, to which 98 (3.1%) declined to participate, leaving the final sample at 3060 university students. The age of the participants ranged from 18 to 54 years (*M*_age_ = 22.7, *DE* = 2.46), and 57.2% of the sample were females (*N* = 1751). Regarding the distribution of the sample across semesters, 7.8%, 9.2%, 18%, 18%, 17.9%, 10%, 7.9%, 5%, 3.4%, and 0.9% of participants were enrolled, respectively, between the first and the tenth semester. Additionally, 0.7% of participants were graduate students and 0.9% did not provide this information. The distribution between sex and semester was homogeneous (χ^2^ = 17.68, *p* = 0.09). 

### 2.2. Instruments

Perfectionism Cognitions Inventory [3]: The 17-item Spanish version of the PCI validated by Esteve-Faubel et al. [18] was employed. This version comprises 6 items capturing PC (e.g., “Why can’t I be perfect?”), 7 items capturing PS (e.g., “I have to work hard all the time.”), and 4 items capturing PD (e.g., “I need to do better.”). Participants were asked how frequently they had thought about these issues in the past week using a 5-point Likert scale of response (see the Introduction section for a more detailed explanation about its psychometric properties). To test the appropriateness of the drafting of the Spanish version of the PCI to the Ecuadorian language, three Ecuadorian psychologists and two Ecuadorian educators examined the wording of the items. No changes were proposed.

Questionnaire about interpersonal difficulty for adolescents (*Cuestionario de Evaluación de Dificultades Interpersonales en la Adolescencia*; CEDIA) [32]: It is a scale that evaluates problems among adolescents from different social contexts. The scale has a multidimensional structure made up of 39 items divided into 5 dimensions: 15 items capturing assertiveness (e.g., “ Do you have difficulty asking a waiter to exchange the cola that you have been served for the orange juice that you had ordered?”), 6 items capturing public speaking (e.g., “Do you have difficulties asking questions in class when you don’t understand what your teacher has explained?”), 6 items capturing heterosexual relationships (e.g., “Do you have difficulties approaching and introducing yourself to someone of the opposite sex?”), 5 items capturing family relationships (e.g., “Do you have a hard time defending yourself when your parents blame you for something you haven’t done?), and 7 items capturing peer relationships (e.g., “Do you have difficulties apologizing to a partner with whom you had an argument?”). Each item is valued using a 5-point Likert-type scale of response (0 = no difficulty; 4 = maximum difficulty).

The original version of the scale showed adequate levels of internal consistency for both the total of the scale (0.91) and all its dimensions (between 0.69 and 0.86) [32]. Furthermore, for the present work, reliability was also acceptable for all its dimensions (α = 0.95, 0.91, 0.89, 0.88, and 0.88, respectively) as well as for the total score of the scale (α = 0.98).

### 2.3. Procedure

A meeting was held with the heads of faculties and degrees to explain to them the aims of this research and to request their cooperation and consent. Participants were informed about the aims of the study. The scales were administrated online using the Google Forms platform so that each participant completed the questionnaire by using their computer or mobile phone during class hours (approximately during 20 min). Ethical standards of the Declaration of Helsinki were followed throughout the procedure. A researcher was present during the administration process to insist on the voluntary and anonymous nature of the activity. Once the tests were completed, participants sent their responses to the final data base. 

### 2.4. Data Analyses

First, Confirmatory Factor Analyses (CFS) were carried out to confirm the factorial structure of the Spanish version of the PCI [18]. Due to the nonexistence of multivariate normality of the data (Mardia coefficient = 190.78), the Satorra–Bentler scaled χ^2^ (S-Bχ^2^) was employed. The following goodness-of-fit indices and interpretation criteria based on Brown [33] and Hu and Bentler [34] were calculated: the Robust Root Mean Square Error of Approximation (R-RMSEA < 0.08), the Standardized Root Mean Square Residual (SRMR: close to 0.08), the Robust Comparative Fit Index (R-CFI ≥ 0.90), and the Tucker Lewis Index (TLI ≥ 0.90). Besides, to choose which model fit the data better, the Akaike’s Information Criterion (AIC) was included (the model with the lowest AIC was preferred to represent the data [35]). Reliability coefficients using Cronbach’s alpha and Pearson’s correlation coefficients between factors were calculated. The magnitude of correlations was interpreted in accordance with Cohen’s [36] criteria: small (*r* = 0.10–0.29), moderate (*r* = 0.30–0.49), and large (*r* ≥ 0.50). 

Subsequently, a Multi-group CFA (MCFA) using S-Bχ^2^ was carried out to verify the factorial invariance of PCI across sex. The stepwise hierarchical method proposed by previous literature was followed [37,38]. First, constraints were imposed to the first-order factor loadings from the base model (model 0) to calculate the first-order metric invariance (model 1). Then, model 1 was compared with model 2, in which second-order factor loadings were constrained. Subsequently, restrictions were established in the intercepts of model 2, creating a scalar or strong invariance (model 3). Likewise, the variances of errors were constrained in model 3 to obtain the strict invariance (model 4). Finally, the variances of first-order factor loadings were constrained in model 3 to obtain the structural invariance (model 5). All generated models should meet the goodness-of-fit indices mentioned before (R-RMSEA, SRMR, R-CFI, and TLI) as well as the level of nonsignificant probability associated to ΔS-Bχ^2^ (*p* > 0.05) [39] and levels of ΔCFI < 0.01 [40]. Additionally, latent mean differences across sex were analyzed using Critical Ratio (CR) statistics and taking into account the suggestions of Tsaousis and Kazi [41], who consider that CR scores > 1.96 or <−1.96 are indicative of the existence of significant differences. The males were the reference group so that they were set to zero when comparing with females. Cohen’s *d* index was calculated to know the size of the differences found considering small (*d* = 0.20–0.49), moderate (*d* = 0.50–0.79), and large effects (*d* > 0.80) [36].

The correlations between the PCI and the CEDIA were observed using Pearson’s product-moment correlation coefficients.

The statistical analyses were performed by using EQS 6.1 (Multivariate Software, Inc., Temple City, CA, USA) and SPSS 22 statistical packages (IBM Software, Armonk, NY, USA).

## 3. Results

### 3.1. Confirmatory Factor Analyses

Based on the 17-item Spanish version of the PCI proposed by Esteve-Faubel et al. [18], several models were tested: a one-dimensional model based on studies that support the one-dimensional structure of the scale [3,13,19,31]; a three-dimensional model which is formed by three independent factors based on the study by Stoeber et al. [31]; a model composed by three independent dimensions, which are correlated to see if a better fit of the data was obtained as stated by Esteve-Faubel et al. [18]; and lastly, a structure of three dimensions encompassed in a higher-order dimension which is a three factor model based on the same latent construct, which complies with the structure of Esteve-Faubel et al. [18]. All these models included the 17 items of the Spanish version of the PCI. The results from CFAs performed can be observed in Table 1. In accordance with the goodness-of-fit indices, only the model with three correlated the factors and the model with three first-order factors and one higher-order factor obtained acceptable levels (R-RMSEA and SRMR < 0.08; R-CFI and TLI > 0.90). However, the model with three first-order factors and one higher-order factor reported the best results for all indices examined and obtained the lowest AIC value. Consequently, the structure of the PCI composed by 17 items divided into three factors: PC (1, 3, 15, 16, 18, and 24 items), PS (9, 12, 13, 14, 17, 23, and 25 items), and PD (2, 4, 5, and 7 items), which are, in turn, encompassed in a second-order factor, was employed for subsequent analysis.

### 3.2. Reliability and Inter-Factor Correlations

Cronbach alpha coefficients were acceptable for the total score of the scale (α = 0.94) as well as its three dimensions: PC (α = 0.87), PS (α = 0.91), and PD (α = 0.86). Positive and significant inter-factor correlations as well as between the three factors and the total score of the scale were observed. All these correlations were of a large magnitude (see Table 2).

### 3.3. Factorial Invariance across Sex

Findings regarding factorial invariance of the PCI across sex are reported in Table 3.

Through the hierarchical method that analyzes the invariance of the PCI, restrictions were included in the model. First, model 0 used as a free model (baseline model) showed adequate fit indices, as did model 1, based on model 0 with constraints in first-order factor loadings (TLI and R-CFI > 0.90; R-RMSEA < 0.05; S-RMR < 0.08; and **Δ**CFI < 0.01). Likewise, in model 2, all second-order factors were restricted, also showing reasonable goodness of fit indices and **Δ**CFI < 0.01 and nonsignificant **Δ**SBχ^2^.

Subsequently, from model 2, restrictions were established in the intercepts of the variables, which gave rise to model 3, which shows adequate adjustment indices and **Δ**CFI < 0.01 and nonsignificant **Δ**SBχ^2^. In the same way, from this model, the error variances constraints were established, giving rise to model 4, with adequate adjustment indices and **Δ**CFI < 0.01 and nonsignificant **Δ**SBχ^2^. Finally, the variances and covariances of the factor constraints were established from model 3, creating model 5, with adequate goodness of fit indicators.

The findings observed in Table 3 show that the factor invariance for model 5 presents adequate adjustment indices (*p* = <0.001, R-CFI = 0.924, R-RMSEA = 0.049 (0.047–0.051), and SRMR = 0.076) and that nonsignificant differences are observed with respect to the comparison of the strong model and the structural model (**Δ**CFI was <0.01 and the **Δ**SBχ^2^ was not significant), which determines that the structure of the PCI is invariant across sex.

### 3.4. Latent Mean Differences across Sex on the PCI

As it has been mentioned in the Method section, males were used as the reference group to establish comparisons based on sex, both for the three first-order factors of the PCI (i.e., PC, PS, and PD) and for the second-order factor (see Table 4). The model statistics for the latent mean structures were acceptable for both the first-order factors (SBχ² = 2216.3740, *df* = 263, *p* < 0.000, R-CFI = 0.923, TLI = 0.911, R-RMSEA = 0.049, CI = 0.047–0.051, and SRMR = 0.075) and the second-order factor (SBχ² = 2305.5103, *df* = 262, *p* < 0.000, R-CFI = 0.919, TLI = 0.906, R-RMSEA = 0.051, CI = 0.049–0.052, and SRMR = 0.070). Nonsignificant latent mean differences were observed across sex for none of the first-order factors of the PCI. In contrast, significant differences of a moderate effect size (*d* = 0.53) were observed for the comparisons between males and females in the second-order factor. Specifically, females scored lower than males.

### 3.5. Correlations between PCI Factors and Interpersonal Relationships

Table 5 shows the results of the correlations between the factors of the PCI and the CEDIA. As it can be seen, all correlations were positive and significant between all the dimensions of the PATs and interpersonal relationships as well as for the total of both scales. All correlations were of a small magnitude, ranging from *r* = 0.06 for the peer relationships and PD and *r* = 0.35 for the total of CEDIA and PC.

Although all these correlations are of a small magnitude, higher correlations between interpersonal relationships and the total PCI as well as PC dimension are observed. In contrast, PD and PS obtained lower, although statistically significant, correlation coefficients with total CEDIA scores and its dimensions.

## 4. Discussion

The aim of this study was to validate a measurement instrument to assess PATs in an Ecuadorian population. Currently, the PCI has only been validated in Canadian [3,19], British [30,31], and Spanish [18] samples. Therefore, although Esteve-Faubel et al. [18] provided a Spanish translation of the scale, this is the first study that has tested its psychometric properties in a Latin American population, such as Ecuador. After carrying out different CFAs to determine which structure was best adapted to the evaluation of PATs in an Ecuadorian population, the results from our study support the structure of the Spanish version of the PCI proposed by Esteve-Faubel et al. [18] composed by 17 items structured into three first-order factors (i.e., PC, PS, and PD) and a second-order factor and evidence that it is a reliable and valid measure to assess PATs in Ecuadorian adolescents.

In the present study, we tried to observe whether the psychometric properties of the PCI in the Spanish language differed between the Spanish and Ecuadorian populations due to cultural diversity as well as the way of administering the scale. It should be remembered that, in the Spanish population, the scale was administered on paper while, in the Ecuadorian population, the scale was administered in an online format. However, it seems that these format changes do not to affect the factor structure and validity of the scale between both Spanish-speaking societies.

Taking into account the interpretation criteria based on Brown [33] and Hu and Bentler [34], this structure, which obtained the best fit to the data, allows to unify the original one-dimensional structure [3,13,19,30] and the multidimensional structure composed by three factors (i.e., PC, PS, and PD) proposed by Stoeber et al. [31]. Thus, the structure of the Spanish version of the PCI confirmed in this study coincides with the multidimensional model in the sense of maintaining the same three factors identified by Stoeber et al. [31] and assumes the existence of a higher-order factor that encompasses these three first-order factors, which is in line with the one-dimensional model defended by Flett et al. [3].

It is important to mention that the multidimensional model of PCI [31] has been criticized by Flett and Hewitt [42] due to the lack of subsequent studies that endorse it, among other reasons. However, Stoeber, Kobori and Brown [43] argued that the three-dimensional structure of the PCI was based on the *Multidimensional Perfectionism Cognitive Inventory* (MPCI) [29], a measure that was created based on PCI. The MPCI measures perfectionist cognitions from dimensions based on personal standards, the pursuit of perfection, and concerns about mistakes. However, research with MPCI is very limited. Hence Stoeber et al. [31] considered reexamining the dimensionality of the PCI, which has greater empirical support. Due to the lack of consensus about the structure of the PCI, Esteve-Faubel et al. [18] tested all of the models previously proposed by literature research, finding better results for a mixed model.

Moreover, the Spanish version of the PCI remains invariant when Ecuadorian females and males are compared. This is an important finding because, from our knowledge, this is the first time that the factorial invariance of the PCI has been tested. With regard to latent mean differences, significant differences were observed only for the second-order factor, with males reporting higher latent means in comparison with their female peers, with moderate effect sizes associated to these differences. This result does not coincide with previous literature that has not found significant differences across sex in the levels of PATs [3,31]. Similarly, our results do not coincide with the study of Downey et al. [22], which obtained a higher presence of PAT in women in comparison with males in the context of eating problems. These differences between our results and previous literature research might be explained because of the different factorial structures of the PCI employed in each of the studies that have analyzed these differences. Unfortunately, as Esteve-Faubel et al. [18] did not provide data about differences across sex, it is not possible to compare our results with previous studies using the PCI structure employed in the current research. Moreover, it is important to mention that this is, from our knowledge, the first study that has analyzed sexual differences based on latent means instead of observed means. Hence, differences between our results and previous literature could be due to the method employed. In this sense, future studies should analyze latent mean differences of PATs in other samples to confirm our results. However, the reason for performing an MCFA using S-Bχ2 to verify the factor invariance of PCI through sex and following the hierarchical stepwise method proposed by the previous literature [37,38] was because, with the progressive restrictions, these analyses allow to check the measurement (model 1), structure (model 3), strict (model 4), and factorial (model 5) invariances.

On the other hand, it is important to consider the link between PATs and the difficulty of social interaction. The findings of the present study show interpersonal difficulties in all dimensions of the PCI as well as for the total score of the scale. In this sense and with all the correlations being significant but of a small magnitude, a higher score of interpersonal difficulties is observed in PC. The PC dimension has fewer social objectives for avoiding being judged and for hypersensitivity to criticism from others [25]. In fact, the PC dimension appears to hinder high-quality friendship relationships [44]. These previous results are in line with those observed in the present study, since, in addition to the lack of assertiveness, it has been observed that public speaking and heterosexual relationships are the highest difficulties for university students with PC, PD, and PS.

It should be noted, on the one hand, that perfectionist traits such as socially prescribed perfectionism are linked to lack of assertiveness [45]. Likewise, it is important to bear in mind that the deficit in social skills is due, in part, to the lack of assertiveness; however, it also focuses on the person’s thoughts and emotions [32]. As it can be seen in the findings of the present study, if these thoughts are related to the need to be perfect, irrational beliefs that originate may hinder interpersonal relationships. Some studies warn of the intensification of social anxiety during the adolescent stage and warn of the fact that growing social demands worry young people about the evaluation that other people have about them [32]. In this sense, the link between PATs and social anxiety [18] and even with hostile behavior [11] is remarkable. Perfectionist self-criticism predicts daily sensitivity to interpersonal sadness, which is understood by the increase in sadness related to the increase, in turn, of a poor and negative social interaction [26].

In this sense, it is important to take into account the model of social disconnection that influences perfectionism. As it has been said before, this model of social disconnection is linked to perfectionist behaviors that hinder interpersonal skills and connection with others. In this sense, the different traits of perfectionism indicate a positive link with aspects such as mistrust, hostility, aggressive and frustrated feelings, and lack of empathy [27]. The social disconnection model warns of the link between perfectionism and personal sensitivity and interpersonal hostility [25]. It is important to keep these findings in mind in order to prevent high PAT among young people, which can complicate their relationships in society in addition to impacting their emotional state.

This study has several limitations. On the one hand, despite the Spanish version of the PCI seemingly being a reliable and valid instrument to assess PATs not only in a Spanish but also in an Ecuadorian undergraduate population, the scale should be employed with caution in other samples. Future studies should test the psychometric properties of this scale in other age samples and Spanish-speaking countries as well as in a clinical population. Similarly, future studies should analyze the temporal stability of the scale and factorial invariance as a function of age. Also, it would be of interest to analyze latent mean differences across different age groups once the factorial invariance across age is confirmed. Another limitation of this study is the fact that the concurrent validity of the scale with other measures of perfectionism or rumination has not been evaluated. However, concurrent validity of PCI has been tested in previous studies that have analyzed the psychometric properties of the scale. Concurrent validity should be observed from scales such as the Mistake Rumination Scale [46], to assess rumination on past mistakes, or the Multidimensional Perfectionism Scale, to assess perfectionist traits such as self-oriented perfectionism, other-oriented perfectionism, and socially prescribed perfectionism [5]. Additionally, it should be noted that, although CEDIA has been used in previous studies with Latin undergraduate students [47], the scale was originally validated in an adolescent sample. Therefore, future studies should test the psychometric properties of the scale in undergraduates. Finally, considering the results about correlations between CEDIA and PCI dimensions, future research might test how much variance of PCI is explained by CEDIA scales through a regression or a SEM model analysis.

## 5. Conclusions

Instead of limitations, this study evidences that the Spanish version of the PCI previously validated in a Spanish sample by Esteve-Faubel et al. [18] can be also used to assess PATs in Ecuador in a reliable and valid way. Before this study, the psychometric properties of the PCI had never been tested in an Ecuadorian sample. This fact reveals, even taking into account cultural diversity, that the previous Spanish translation is adaptable to this country, which has, from now on, a specific measurement scale of PATs. This scale represents a combination of the different structures, one-dimensional [3,13,19,30] and multi-dimensional [18,31], proposed by previous literature and allows to evaluate the PAT as a whole and more specifically by using each of its factors, which delimits a greater concretion in the reflections between PC, PD, and PS. This detail further specifies how the minds of subjects with PATs work, and it opens up a greater range of study and prevention in the field of psychology and education. From this knowledge we will be able to mainly concretize its treatment and its prevention.

Moreover, because research about PATs has been conducted on western samples, this study is a further step in scientific research about PATs, as it allows to expand research about this construct in Latin America. Considering the close relationship between PATs and psychopathology (e.g., 3, 11, 15, and 17) as well as the relation with difficulties of social interaction, it is important to know its possible consequences in collectivistic cultures, such as Latin American societies. Having validated the PCI in an Ecuadorian population, further research is expected to prevent maladaptive patterns of perfectionist rumination.

## Figures and Tables

**Table 1:** Goodness-of-fit indices for the different models based on the Spanish version of the Perfectionism Cognitions Inventory (PCI) in an Ecuadorian population.

	S-Bχ²	χ²	df	AIC	R-RMSEA 90% CI	SRMR	R-CFI	TLI
Model with one factor	3934.39	7075.72	119	3696.39	0.102 [0.100, 0.105]	0.082	0.848	0.826
Model with three non-correlated factors	5970.63	9274.45	119	5732.63	0.127 [0.124, 0.130]	0.370	0.766	0.733
Model with three correlated factors	2258.21	3827.83	116	2026.212	0.078 [0.075, 0.080]	0.065	0.914	0.900
Model with three first-order factors and one higher-order factor	1596.65	3905.72	116	1364.65	0.065 [0.062, 0.067]	0.064	0.941	0.931

*Note*: *p* < 0.001 for S-Bχ² and χ² in all cases. S-Bχ² = Satorra–Bentler χ² scaled; df = degrees of freedom; AIC = Akaike’s Information Criterion; RMSEA = root mean square error of approximation; CI = confidence interval; SRMR = standardized root mean square residual; CFI = comparative fit index; TLI = Tucker Lewis Index.

**Table 2:** Means, standard deviations, and Pearson’s correlations.

Subscale	PC	PS	PD	*M*	*SD*
PC	1			10.15	4.97
PD	0.59 **	1		8.19	3.64
PS	0.72 **	0.79 **	1	13.83	6.14
Total	0.87 **	0.86 **	0.95 **	32.18	13.27

*Note*: *p < 0.005; ** *p* < 0.01; PC = Perfectionist Concerns; PS = Perfectionist Strivings; PD = Perfectionists Demands.

**Table 3:** Goodness-of-fit indexes for the two-factorial model of the PCI depending on sex.

	χ^2^	S-Bχ²	df	TLI	R-CFI	R-RMSEA	SRMR	ΔS-Bχ² *(*Δdf*, p)*	ΔR-CFI
Males	1619.08	652.40	116	0.945	0.953	0.059 [0.055, 0.064]	0.059		
Females	2488.44	1034.14	116	0.921	0.933	0.067 [0.063, 0.071]	0.070		
Model 0	4107.52	2106.57	232	0.913	0.926	0.051 [0.049, 0.053]	0.065		
Model 1	4127.12	2180.47	246	0.915	0.923	0.051 [0.049, 0.053]	0.066	20.70 (14, 0.110)	−0.003
Model 2	4134.75	2154.09	249	0.917	0.924	0.050 [0.048, 0.052]	0.075	1.86 (3, 0.603)	0.001
Model 3	4146.56	2229.83	266	0.910	0.923	0.049 [0.07, 0.051]	0.075	12.03 (17, 0.798)	−0.001
Model 4	4165.31	2247.54	283	0.913	0.924	0.048 [0.046, 0.049]	0.075	10.68 (17, 0.873)	0.001
Model 5	4148.57	2212.64	268	0.912	0.924	0.049 [0.047, 0.051]	0.076	0.52 (2, 0.773)	0.001

*Note:* Model 0 = free model; Model 1 = model 0 with first-order factor loadings; Model 2: model 1 with second-order factor loads; Model 3 = model 2 with intercepts; Model 4 = model 3 with error variances; Model 5 = model 3 with variances and covariance factors; PCI = Perfectionism Cognitions Inventory; S-Bχ² = Satorra–Bentler χ^2^ scaled; df = degrees of freedom; TLI = Tucker Lewis Index; R-CFI = Robust Comparative Fit Index; R-RMSEA = Robust Root Mean Square Error of Approximation; CI = confidence interval; SRMR = Standardized Root Mean Square Residual; ΔS-Bχ² = χ² difference model comparison test; Δdf: difference between degrees of freedom, ΔR-CFI = Robust Comparative Fit Index difference test.

**Table 4:** Latent means differences across sex in the PCI.

	PC	PS	PD
**First-Order Factors**			
Males (reference)			
Females			
Mean estimate (ME)	−0.003	−0.008	0.018
Standard error (SE)	0.024	0.0033	0.029
Critical Ratio (CR)	−0.129	0.246	−0.614
*D*	-	-	-
**Second-Order Factor**			
Males (reference)			
Females			
Mean estimate (ME)	−0.911		
Standard error (SE)	0.064		
Critical Ratio (CR)	−14.128 *		
*D*	0.528		

*Note*: PC = Perfectionist Concerns; PS = Perfectionist Strivings; PD = Perfectionist Demands; *: Statistically significant difference (>1.96 or <−1.96).

**Table 5:** Correlations between PCI factors and interpersonal relationships.

	PC	PD	PS	PCI Total
Assertiveness	0.25 **	0.19 **	0.20 **	0.28 **
Public speaking	0.33 **	0.23 **	0.21 **	0.28 **
Heterosexual relationships	0.28 **	0.31 **	0.27 **	0.32 **
Family relationships	0.33 **	0.08 **	0.12 **	0.20 **
Peer relationships	0.32 **	0.06 **	0.11 **	0.19 **
Total CEDIA	0.35 **	0.21 **	0.21 **	0.29 **

*Note*: * *p* = < 0.05; ** *p* = 0.01; PC = Perfectionist Concerns; PS = Perfectionist Strivings; PD = Perfectionist Demands.
